# Refining Procedures within Regulatory Toxicology Studies: Improving Animal Welfare and Data

**DOI:** 10.3390/ani11113057

**Published:** 2021-10-26

**Authors:** Helen Prior, Hollie Blunt, Lee Crossman, Aidan McGuire, Ruth Stow, Fiona Sewell

**Affiliations:** 1 National Centre for the Replacement, Refinement and Reduction of Animals in Research (NC3Rs), London NW1 2BE, UK; fiona.sewell@nc3rs.org.uk; 2 Sequani Limited, Ledbury HR8 1LH, UK; hollie.blunt@sequani.com; 3 Labcorp Early Development Laboratories Ltd., Harrogate HG3 1PY, UK; lee.crossman@labcorp.com (L.C.); ruth.stow@labcorp.com (R.S.); 4 Charles River Laboratories, Edinburgh EH33 2NE, UK; aidan.mcguire@crl.com

**Keywords:** 3Rs, fasting, metabolism, microsampling, non-rodents, refinements, rodents, social-housing, toxicology

## Abstract

**Simple Summary:**

Before any new medicine can be administered to humans, or new chemical marketed, some tests using animals such as fish, mice, rats, rabbits, dogs, or monkeys are performed in order to satisfy the legal requirements of international regulatory and government agencies. These assess the potential for harmful side effects in humans or species found in the environment and to explore how the compound is processed within the body. The housing conditions and procedures performed (such as dosing of compounds and removal of small blood samples) are designed to minimize any pain, suffering, distress, or lasting harm that may be experienced by the animals. These refinements improve animal welfare but can also improve the data quality. Examples of new processes, technologies, or equipment that have been introduced within some UK facilities are shared in this article and provide opportunities to benefit many more animals undergoing testing across the world in the future.

**Abstract:**

During the development of potential new medicines or agrochemicals, an assessment of the safety profile to humans and environmental species is conducted using a range of different in silico and in vitro techniques in conjunction with metabolism and toxicity studies using animals. The required studies are outlined within international regulatory guidelines which acknowledge and support the application of the 3Rs to reduce the number of animals used or to refine the procedures performed when these studies are deemed to be necessary. The continued development of new technologies and adoption of best-practice approaches to laboratory animal housing and study procedures has generated a series of refinements that can be incorporated into animal studies throughout the package. These refinements benefit the welfare of fish, mice, rats, rabbits, dogs, minipigs, and non-human primates (NHPs) whilst maintaining or improving data quality within general toxicology, metabolism, and other studies and can also bring efficiencies to processes that benefit study costs and timings. Examples are shared which cover the following topics: social housing of dogs and NHPs, surgical refinements in the rat bile duct cannulation model for collection of data for metabolism studies, whether fasting is really required prior to clinical pathology sampling, and the use of microsampling for toxicokinetics.

## 1. Introduction

During the development of potential new medicines and agrochemicals, an assessment of the safety profile to humans and environmental species is conducted. In silico and in vitro techniques are used where available and applicable, and the field has expanded rapidly to investigate, develop, and validate alternative non-animal methods over the last decade [1,2]. However, studies using animals still underpin the current requirement to provide data supporting first-in-human trials and marketing for pharmaceuticals and for risk/hazard assessment of chemical products across the world. The required studies are outlined within international regulatory guidelines [3,4] to investigate general and reproductive toxicity, genotoxicity, safety pharmacology, and carcinogenicity risk and to understand metabolic profiles where appropriate to humans and environmental species. The guidelines acknowledge and support the application of the 3Rs to reduce the number of animals used or to refine the procedures performed, when studies using animals are deemed to be necessary to obtain the data required (i.e., when replacement of animal studies is not yet possible).

Within the United Kingdom, the animal studies that are conducted for metabolism and toxicity assessments are authorized under the governance of the home office in compliance with local and regional animal welfare laws (i.e., Animals (Scientific Procedures) Act 1986 and EU Directive 2010/63/EU 2013 [5,6]). The National Centre for the Replacement, Refinement, and Reduction of Animals in Research (NC3Rs) is an independent scientific organization established by UK government to support scientists to discover, develop, and promote new ways of applying the 3Rs to animal use. The principles of refinement use housing and husbandry practices and experimental procedures that minimize the pain, suffering, distress, or lasting harm that may be experienced by research animals to improve their welfare. There is a growing evidence base showing that pain and suffering can alter an animal’s behavior, physiology, and immunology, leading to increased variation in experimental results which, in turn, can impair both the reliability and repeatability of studies. Thus, improvements to animal welfare can also improve the quality of science [7]. Many animal welfare initiatives and refinement activities led by the NC3Rs, and other 3Rs centers and consortia, are equally applicable to animals used for toxicology and metabolism assessments. The NC3Rs also directly supports the toxicology community via long-standing collaborations with the Association of British Pharmaceutical Industry (ABPI), European Centre for Ecotoxicology and Toxicology of Chemicals (ECETOC), other industry consortia and societies or individual companies, with office-led projects and meetings, workshops and conference sessions to investigate opportunities to apply the 3Rs within the different toxicology studies across the sectors. The sharing of data and experience between multiple companies have identified best-practices in processes or procedures that promote refinements such as social housing during telemetry recordings [8], microsampling for toxicokinetic (TK) blood sampling [9], multi-faceted improvements to bile duct cannulation studies [10], and adoption of evident toxicity for acute inhalation toxicity studies [11]. Some of these topics are expanded further with current industry data in the following sections. The CRACK IT open-innovation platform [12] has facilitated provision of new products that refine current/previous procedures in response to industry requests, such as home cage monitoring for rodents [13] and wireless neural recording systems for mice [14].

Refinements for toxicology and metabolism studies can apply to specific species, procedures, or housing conditions, and therefore encompass a wide range of potential improvements for animal welfare and data. A summary of general principles, which are directly applicable to toxicology and metabolism studies in the following ways, is highlighted below.

Replacing death as an endpoint with use of evident toxicity: older guidelines for acute toxicity studies often rely on identification of the LD_50_ (the lethal dose for 50% of the animals). Recognizing that death as an endpoint is likely to be preceded by severe pain and/or suffering, earlier humane endpoints and the concept of evident toxicity are accepted within new and refined guidelines for acute inhalation (OECD TG 433; [4,11]), oral (OECD TG 420 [4]), and dermal studies (OECD TG 402 [4]). Evident toxicity is the presence of clinicals signs that predict severe toxicity or death in most animals at the next highest concentration or dose of the chemical. These principles are a refinement since the next (higher) concentration/dose level need not be performed (also reducing animal use). More recently, work has begun to standardize recording of sublethal clinical signs of toxicity in fish, aiming to move away from LD_50_ and mortality as the endpoint for acute fish studies (OECD TG 203; [4,15]).

Use of appropriate (high) dose levels: investigating and identifying potential target organ toxicities and dose response relationships may result in animals experiencing adverse symptoms or toxicities following dosing. Criteria for defining the maximum tolerated dose (MTD) in short term pharmaceutical studies (up to seven days in duration), using bodyweight loss of 10% in rats, dogs, and non-human primates (NHPs) [16] or a combination of mild clinical signs and 5% bodyweight loss in rats [17] minimize the potential for suffering and act as early toxicity markers. Guidance on dose level selection for general toxicology studies is available for pharmaceuticals [18]. More recently, a thorough review of dose level selection and application relevant for all chemicals has been published [19], aiming to update and harmonize global recommendations and balance untoward animal suffering.

Training/acclimatization for invasive procedures: adequate socialization and habituation to technicians and care staff, and training for cooperation with husbandry and experimental procedures can reduce stress and/or time for procedures to be conducted. Examples of procedures where training could be beneficial within the toxicology field include chair-restraint for NHP dosing, blood sampling or electrocardiogram (ECG) collection [20], sham oral dosing of dogs [21], wearing of jackets for intravenous infusions or external ECGs in dogs and NHPs [8], wearing of face masks for inhalation dosing in dogs, and NHPs and acclimatization of rats to restraint tubes for head-out plethysmography [22] or inhalation dosing.

Housing and husbandry practices: animals on toxicology and metabolism studies can benefit from best-practices such as above-minimal space per animal, social housing (see below) and provision of environmental enrichment. Information on refined techniques such as non-aversive methods for handling mice, rat ticking/playtime, and opportunities to refine the use of NHPs are provided on the NC3Rs website [23].

Social housing as a form of environmental enrichment: the presence of compatible companions within the home cage/pen is a fundamental source of environmental (social) enrichment and allows natural behaviors to be expressed (for example, grooming, playing). Reducing any required periods of separation from cage/pen mates during a study to the minimum required for the study objectives is a positive refinement. For example, recording of dog or NHP ECGs via jacketed telemetry within the home pen, instead of removing the animal to a separate room for ‘snap-shot’ ECGs (the telemetry technique also provides more and higher quality data). Recordings from surgically-implanted telemetry devices can now be made from multiple animals within the same cage/pen for both rodents and non-rodents, reducing the need for individual housing during safety pharmacology and toxicology studies collecting cardiovascular and other data [24,25]. Rats, dogs, and NHPs are also isolated for collection of urine and feces for metabolism studies. These too can be performed in group housed conditions [26,27] and are described further in Section 2.

Refinements for surgical procedures: these include the appropriate use of anesthetics and analgesia, procedures or techniques that shorten surgery time or improve success rates, appropriate recovery periods etc. Typical surgical procedures within toxicology studies include implantation of telemetry devices [28], bile duct cannulations [10], vascular access ports, or indwelling catheters for repeat intravenous doses or blood samples.

Using a pre-existing procedure for an additional purpose: for example, microchip transponders are often implanted for identification purposes, yet a similar product with additional functionality implanted in a different area of the body can be used for body temperature measurements [29], and/or to track the position of the animal as part of locomotor activity assessments [13], with remote or cage-side non-invasive measurements in both cases. Another example is the use of a jacket for external ECG recordings from dogs or NHPs which can incorporate respiratory measurements by inductive plethysmography bands within the same jacket.

Replacing invasive procedure(s) with non-invasive: for example, body temperature measurement with a rectal probe is an invasive procedure, in comparison with surface temperature measurements via infra-red camera or hand-held device.

Taking smaller samples: more sensitive analysis methods mean that smaller samples are needed, which can reduce the time taken for the procedure to be performed. For example, as the bioanalytical community has adopted use of more sensitive Liquid Chromatography with tandem mass spectrometry (LC-MS/MS) equipment, smaller blood ‘microsamples’ can be collected, with less of an effect on overall blood volumes in rodents (see Section 4 for further details). If blood taken for other analyses, such as for the various parameters within hematology and clinical pathology panels, were also able to be analyzed via newer/more sensitive equipment or tests, then the potential purposes for microsampling would widen and smaller samples would be possible throughout a toxicology study.

The following examples need careful consideration as to whether the definition of refinement can be correctly applied. Obtaining more data from the same animal, by inclusion of additional non-invasive techniques or observations, allows for direct comparison of different data within the same individual. However, it is not a refinement. If this avoids the use of separate animals to provide this data, this is a reduction in overall animal use. An example of this is microsampling, where repeated small samples may be taken from an individual mouse, instead of using more animals (see Section 4 for more details), or inclusion of safety pharmacology endpoints (functional observational batteries, respiratory counts, cardiovascular data) into toxicology studies [30]. The balance between any potential pain or suffering due to surgical procedures (e.g., cannulations for intravenous dosing, telemetry device implantation) and the welfare benefits that may result from these (i.e., reduces overall number of procedures when repeat dosing is required, non-invasive collection of cardiovascular data) needs to be the topic of discussion for ethical boards/panels regarding the lifetime experience of the animals.

The following sections provide details of some refinement opportunities developed within UK organizations, with the aim of sharing this information to encourage discussion and uptake within other facilities and to apply the 3Rs to more animal studies used for toxicology and metabolism purposes.

## 2. Refinements to Dog and NHP Caging for Metabolism Studies (Ruth Stow)

Metabolism cages are designed to enable the collection of urine and feces from animals during the conduct of absorption, distribution, metabolism, and excretion (ADME) studies, enabling an ‘excretion balance’ assessment to be made as part of the package of data required for regulatory submission of novel pharmaceuticals and chemicals. Historically, the design of either dog or NHP metabolism cages has involved single housing; these conditions during collection periods have limitations for normal social behaviors in both species. Additionally, due to their dimensions, metabolism cages significantly limit normal vertical movement in the NHP (note that height of the cage or enclosure is particularly important for NHPs which flee upwards when alarmed).

A recent (2016–2021) joint collaboration between Labcorp Harrogate (research facility in UK) and Novo Nordisk A/S (Sponsoring company in Maaloev, Denmark), has challenged and refined the current single housing of both dogs and NHPs for ADME studies [26,27]. The aim of these refinements was to reduce the need for single housing and the stress that results from it and therefore decrease the welfare impact of metabolism cages on laboratory dogs or NHPs whilst generating ADME data for regulatory submission.

**Figure 1:**
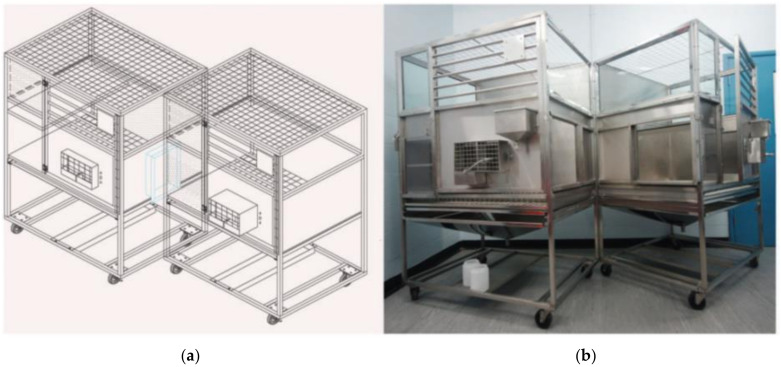
Schematic (**a**) and photo representation (**b**) of the dual cage system with a hatch between the two connected cages for pair-housed dogs.

The refinements to dog metabolism cages have been basic but effective, using the existing cages with some simple modifications. By combining two (or three) metabolism cages with the introduction of a sliding hatch incorporated into the side of each cage, this has allowed either paired or triple housing (Figure 1). Additional modifications included an adjustable removable shelf to allow height adjustment for smaller dogs and replacement of the stainless-steel front side panel with a clear Perspex panel to improve visibility for the dogs. These refinements have enabled mass balance study objectives to be achieved, whilst improving the animal welfare, with anecdotal evidence from animal technicians that animals were much calmer as a consequence of paired or group housing. The cages can still enable animals to be separated if required, for example during blood collection, sample collection during cage cleaning and during feeding.

**Figure 2:**
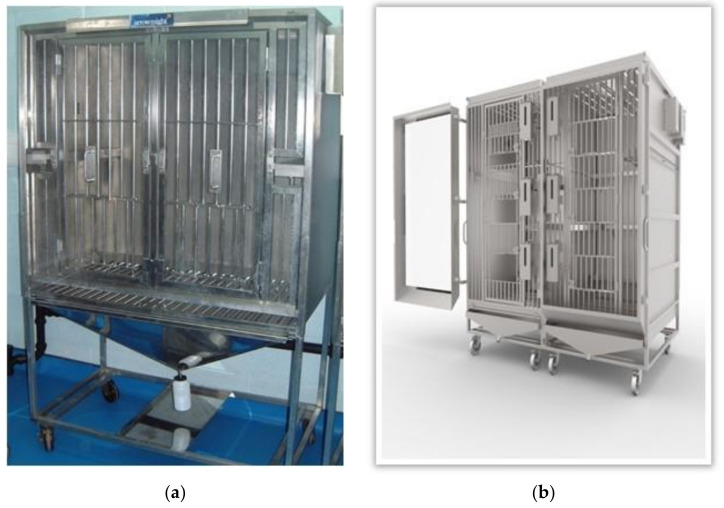
Metabolism cages for single (**a**) and group (**b**) housing of NHPs.

Modifications to the NHP caging, by contrast have been radical, involving the complete redesign of the cage. The dimensions of a traditional single-housing NHP metabolism cage (see Figure 2a) are typically 100 cm high × 60 cm deep × 110 cm wide. This cage contains a roll bar style open flooring to allow the recovery of urine and feces below the cage unit, and internal shelves for perching at the side of the cage. The low height of the cage allows only very restrictive ‘fight or flight’ response to the back of the cage. The cage has sub-optimal lighting and air flow and it is difficult for laboratory staff to handle animals in the cage due to the ‘squeeze-back’ mechanism of action. Throughout the study period, a Perspex^®^ screen was placed over the front bars to prevent the animal urinating outside the cage, further restricting air flow within the cage.

The dimensions of the newly designed group-housing cages (Figure 2b) are 187.5 cm high × 118 cm deep × 200 cm wide, which is more than double the volume per animal of the single-housing cages. Improvements include a Perspex^®^ roof with a slotted ceiling panel to allow greater light but also an additional benefit of providing enrichment games, as small food enrichment items and ping pong balls can be placed on top of the cage roof for the animals to play with. The increased height (almost twice that of single housing cages) provides the NHP with the opportunity to escape to higher vantage points within the cage, swing bars and shelves at different heights allowing variable views, group hierarchy and flight response to be maintained, and to further facilitate the normal flight response, escape runs were incorporated between the two halves of the cage, at two different levels.

**Figure 3:**
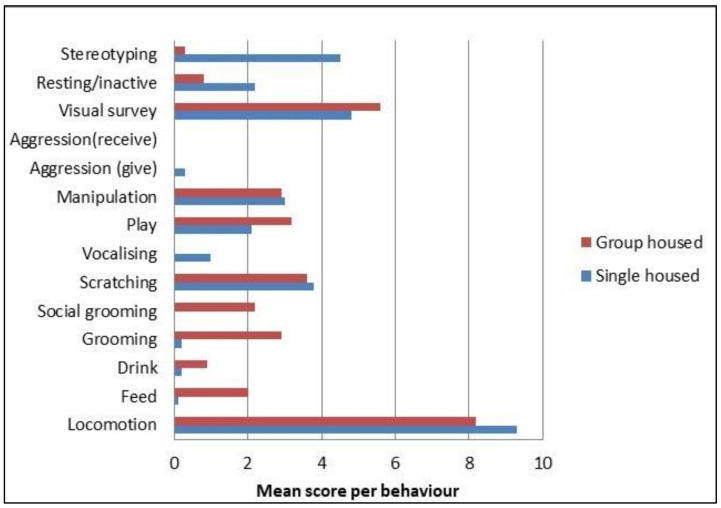
Mean number of observations for typical NHP behaviors over a seven-day observational period for single- and group-housed (*n* = 3) NHPs (further details on the validation study and results are provided within [27]).

The new cage also incorporates features to facilitate quicker/easier handling of the animals, such as a ‘pull forward’ capture mechanism, rather than a ‘squeeze back’ system and natural divisions via shelving and sliding panels to enable animal segregation for limited periods (e.g., for blood sampling). These allow for calmer procedures and potential reductions in stress (for both NHPs and animal technicians). Indeed, during validation of the new cages, recording of behavioral observations indicated that group housing has a beneficial impact, with increased play and grooming and the absence of stereotyping (e.g., head turning, rocking, pacing, or aggression) behaviors compared with single-housed animals (Figure 3).

Improvements for sample collection (and hence data accuracy) have also been incorporated, with modified shelving to prevent any seepage, drainage gaps between shelves and cage walls, and angled shelves to facilitate drainage of urine and cage washing. During validation of the new cages, the total mean recoveries of radioactivity in excreta from group housed NHPs were equivalent to the recoveries achieved from single housed NHPs and also consistent with the historical single housed data. Recoveries were within the individual variability range of 10% [27].

The larger cages described here for dog and NHP metabolism studies allow for group housing of animals for the majority of the study period, in compliance with UK and European expectations of these conditions as standard [6]. Experience gained over three years of metabolism studies in group housed cages has indicated a positive welfare impact for the study animals with no detrimental effect on the data collected. The lack of individual data within study reports has not been questioned, with pooled cage data accepted as a consequence of the improved animal welfare. Use of the group housing cages is therefore the preferred method for ADME studies generating data for regulatory submission and logistics for expansion of similar caging across other Labcorp sites in the UK and USA is being explored.

## 3. Refinements in the Bile Duct Cannulated Rat Model (Lee Crossman)

Rats are typically the rodent species of choice for investigating the ADME of novel pharmaceuticals and agrochemicals, with studies required for regulatory approval of new products. Since rats lack a gall bladder, they are an ideal model for investigating continuous biliary excretion and biotransformation which contributes to understanding of first-pass metabolism. The bile duct cannulation (BDC) model has previously been a focus for collaborative refinements between UK organizations [10], with a modified tail cuff and cannula system (hereby described as the pin-port model) designed and introduced at Labcorp. This facilitates use of the dual-cannulation method which allows recirculation of bile back into the duodenum during the period following surgical cannulation or wash out periods and simultaneous collection of bile and reinfusion of bile salts following dose administration. This innovation means that animals do not require tethering or single housing during periods where bile is not being collected. See Appendix A for further details of the surgical and study processes.

**Figure 4:**
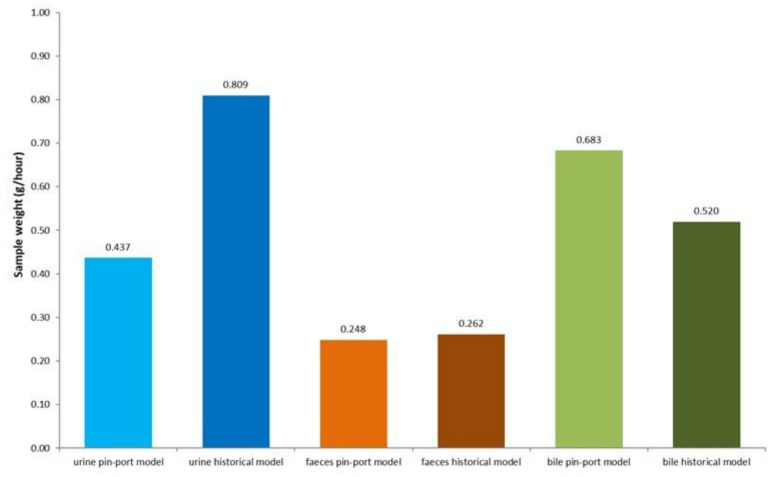
Mean excretory sample weights (g) from five collection periods (see Appendix A for study design) compared to ten studies using the previous BDC (historical) method. Sample weight data from all five sessions were totaled for individual animals and the mean data for the six rats are presented.

**Figure 5:**
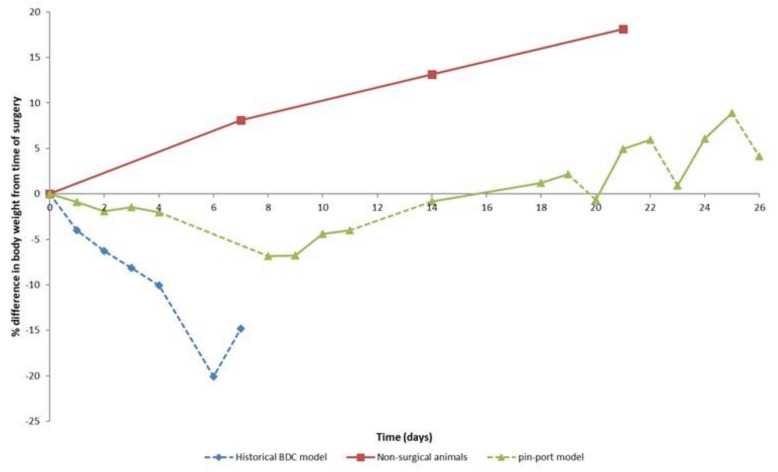
Bodyweight/growth comparison between non-surgical animals and animals that had received BDC surgery that were either singly housed and tethered (historical BDC model) or group housed and untethered during the recovery period (pin-port model). Dashed lines indicate when animals were housed singly in metabolism cages. Non-surgical animals Day 0 taken as time when animals were of similar age and weight as surgical animals at the time of surgery.

A validation study was run using six male rats (Han Wistar, 320–345 g at time of surgery), with animals placed singly in metabolism cages during collection periods and group housed in standard caging during post-surgical recovery and between sampling periods. The study was designed to mimic regulatory BDC studies using an oral dose (sample collection phase 1) and an intravenous dose (sample collection phase 2). A further three sample collections (phases 3–5) without dosing and designed to mimic a screening program were also conducted (see Appendix A for further details of the study design). Surgical success rates, animal health observations, bodyweight losses, excretory output, and bile flow were compared to historic data from animals which were tethered and singly housed during the entire period (Figure 4 and Figure 5).

The surgical success rate of the modified (pin-port) method was 83%, which was comparable to historic success rates of 89%. Slightly lower success rates using the pin-port method were due to the cannula(e) detaching from the pin-port and further modification using additional sutures has since eliminated this issue. No observations of animals chewing the cannulae and preventing bile from being collected were observed using the pin-port method, which had been the major reason for early termination in the historical method. The only observation was local irritation to tail cuff, with no other behavioral or clinical signs noted. Bile duct cannulae remained patent over the 26-day study period and excretory outputs and bile flows were acceptable to support investigations into routes and rates of excretion. Mean bile flow in pin-port animals (0.683 g/h) was elevated in comparison to the historical method (0.520 g/h). Urine volume was lower in the pin-port model (0.437 g/h), this was a likely consequence of animals in the historical model being given lectade which resulted in higher urinary output (0.809 g/h). Fecal output was comparable between models (0.248 g/h pin-port model, 0.262 g/h historical model).

Bodyweight losses in the post-surgical period from animals group housed post-surgery were <2%, which was five-fold lower compared to animals tethered and singly housed immediately post-surgery (Figure 5). Bodyweight losses following periods of confinement/tethering in a metabolism cage were ≤10% in the pin-port animals and ≤20% in the historical method. Bodyweight rose rapidly following removal from the metabolism cage, untethering and group housing (around 2.5 g/day), with growth curves consistent to non-surgical animals (around 2.7 g/day), suggesting bodyweight losses in the pin-port group are linked to tethering and single housing rather than surgery or analgesia.

Surgical success rates, reduction in body weight losses, comparable animal health observations, and acceptable excretory output and bile flow results all suggest the pin-port method improves animal health and welfare without infringing scientific integrity. It also offers the potential to reduce the number of animals undergoing surgery to support metabolism studies. On this basis the pin-port model is the only model in use at Labcorp UK sites in support of BDC studies, and the historical method is no longer offered.

## 4. Microsampling (Hollie Blunt)

The advent and implementation of microsampling has radically changed the landscape of in vivo nonclinical toxicology studies over the last decade, fulfilling multiple components of the 3Rs and improving the quality of the scientific data obtained [9]. The approach of collecting small biological sample volumes (typically ≤ 50–100 µL), for analyses such as exposure assessment to a test substance, initially focused on the ability to reduce animal use compared with studies that required conventional sample volumes (>0.2 mL). Such opportunities have significantly reduced rodent use over recent years, particularly for studies with juvenile animals [31]. Since then, the concept has evolved to bring additional benefits to the forefront, including refinements to the blood sampling techniques themselves and increasing in the power of the scientific data obtained.

Collection of smaller sample volumes allows the use of less invasive sampling techniques, which benefit rodents in particular in the following ways. Microsamples are readily obtained from superficial veins such as the lateral tail vein, without the need for warming for vasodilation nor the need for anesthesia, which is used by some laboratories for alternative sampling routes (e.g., sublingual vein sampling). This thereby reduces the impact on an animal’s condition, allowing a rapid recovery and reduces time away from the home cage environment and companions. Techniques that do not require the use of anesthesia are also particularly valuable in studies with pregnant females, where its use can cause inadvertent fetal death. Although rodents are the primary benefactors of such techniques, refinements for non-rodents such as the NHP, dog, minipig and rabbit have also been established. Circulatory blood volumes are not usually limiting factors in these species, but the ability to collect samples from superficial veins (such as the ear vein in minipigs) and the shorter time required also significantly improves their experience of the sampling procedure.

Scientifically, microsampling has improved the quality of data obtained in a number of ways. In the case of TK assessments in rodents, the technique can allow the collection of a full kinetic profile from each animal in a small satellite group, thereby providing individual animal exposure data for review (serial sampling, as per Table 1). Conversely, nonclinical studies can be designed without the need for designated satellite animals, with the samples obtained from the animals assigned to toxicity assessment instead (main study animals). Under this approach, the samples are distributed across all of the available animals, thereby reducing the number of collection points from each individual, whilst still providing exposure data for the overall study population (composite sampling, as per see Table 1). Such approaches are now actively encouraged within regulatory guidance (ICH S3a Q&A), due not only to the animal welfare benefits, but also the ability to provide a snap-shot of exposures for each individual animal. This allows for the correlation of clinical findings with exposure in individual animals (a possibility that had not been available before). With careful study design, the collection of blood samples for TK in this way, does not significantly impact on other important study end points, such as clinical pathology [32].

The advantages of microsampling do, however, require careful balance and consideration, to ensure the initial benefits are not offset by unintentional harms. It is often perceived that microsampling allows more samples to be collected per animal without exceeding permitted blood volume withdrawal limits, but this increases the number of blood sampling procedures (‘needlesticks’) each individual animal experiences. This is a vital consideration within any study design and a wider discussion on the maximum acceptable tolerances of this is encouraged. Furthermore, the anticipated toxicity profile of a test substance should be understood so that the blood sampling strategy can be defined before pivotal nonclinical studies take place. This is to ensure that the resultant side effects are compatible with the adopted blood sampling approach. For example, if hematology changes such as anemia are anticipated, then it may not be appropriate to collect a high number of blood samples per animal (and composite designs may be more appropriate). This consideration can be accounted for by the conduct of well designed, targeted dose range-finding studies, which in turn will confirm the most appropriate microsampling approach to be used. The ability to tailor the microsampling approach to the needs of each study shows its continued strength for advancing the 3Rs and will continue to support the safe development of medicines, food additives, chemicals, and crop protection products for the future.

## 5. Is fasting Required before Clinical Pathology Bleeds on Toxicology Studies? (Aidan McGuire)

The collection of blood samples to assess test article effects on clinical chemistry and hematology is a routine procedure within toxicology studies, and provision of this data is a standard requirement within most studies for new pharmaceuticals and agrochemicals during development. Historically, it has been common to fast rats and non-rodents (such as dogs, minipigs, and non-human primates) before these procedures. This ensures a standard baseline to allow even comparisons between animals and over the time period of the study, and to mimic clinical sampling conditions. Whilst there are no regulatory requirements which stipulate the need to fast animals, it is recommended in some older chemical guidelines [4] and guidance from a joint taskforce of toxicological pathologists [33]. The minimum fasting period is generally accepted as 12–18 h, typically achieved by removal of food overnight with blood sampling the next morning. This can affect the different laboratory species in different ways. Dogs and minipigs are generally fed during the day and eat their whole ration in a short period. Therefore, they are not intentionally fasted before morning bleeds and normal routines are maintained. NHPs generally have unlimited access to food and since much of the portion is provided as graze on the pen floor, intentional fasting is not practical to achieve. However, as NHPs generally consume small amounts of food throughout the day and normally sleep during the night (the immediate period before blood sampling), they are assumed to be near-fasted. Overnight fasting in rodents, who are active during the night and mainly feed during this time, is more likely to have an impact on these species; the fast may even be longer than intended for individual animals if they have not fed during the preceding daylight hours. Due to small bodyweights and blood volumes of mice, clinical pathology samples are generally not collected from this species (other than at necropsy) and fasting is therefore not required. Consequently, it is really only rats that are intentionally fasted before sampling.

A number of different clients placing studies at the CRL Edinburgh facility do not require fasting of rats prior to clinical pathology bleeds on their toxicology studies, considering this as an outdated practice. Whilst this simplified husbandry and technical procedures, it meant different procedures were conducted within the facility for the same ‘typical’ studies for different clients. However, this did provide opportunities for qualitative assessment of the advantages and disadvantages of fasting and initial reviews of the data indicated that interpretation was unaffected, as most comparisons were made with the contemporaneous control groups that had followed the same process (either fasted or non-fasted). In 2010, the CRL Edinburgh facility conducted an internal validation study to formally evaluate the effects of fasting on clinical pathology blood results in rats and use this data to justify the non-fasting of rats when requested.

The validation study consisted of two groups of Sprague Dawley rats and a further two groups of Han Wistar rats (*n* = 20, 10 males and 10 females per group, of around 9 weeks old at the first sampling occasion). During Week 1, one group per strain was fasted prior to clinical pathology blood sampling (approximately 17–18 h without food, but with access to water), whilst the other group per strain were not fasted. A second blood sample was taken five weeks later with the fasting regime reversed. Hematology, plasma clinical chemistry and coagulation assessments were made. As data were consistent between the strains, males and females, only a summary for male Sprague Dawley rats and selected parameters are presented (Table 2).

The only major differences between the data were lower glucose and triglyceride levels in fasted animals. All other differences were considered minor and/or within natural variation. There was no evidence that the individual data were less or more variable after fasting compared to non-fasting and the same findings were evident in both strains and both sexes. Following the completion of this study, intentional fasting before clinical pathology blood sampling was stopped for rats, in line with other species, for all studies and sponsors.

More recently, a comparison was made between historical control data for cynomolgus monkey studies between the UK site (where the animals are not fasted) and a North American site (where the animals are fasted overnight). Only data for the previous two years clinical pathology parameters were compared, as both sites used the same analytical equipment and assays. However, animal supply, housing, acclimatization periods, and most importantly blood vector (plasma in UK versus serum in USA) were different between the sites. When the data were converted to the same units it showed similar findings to that observed with the previous rat validation study. The only major differences between the data were lower glucose levels in fasted animals (Table 3). There was no clear evidence that the individual data were less or more variable after fasting or non-fasting and the same findings were evident in both males and females.

We conclude that there is no clear evidence that fasting reduces variability in the clinical pathology and hematology data in the toxicology species examined, and as data are compared with contemporaneous control values, any differences in glucose and triglyceride levels from ‘historical fasted data’ has no overall impact on interpretation within a single study. Removing the requirement for fasting is a significant refinement, especially for rodents, but also reduces technician time and interruptions of normal procedures within each study (as food hoppers do not need to be removed and recorded). Accordingly, this refinement was also adopted prior to necropsies of all species, with small adjustments easily made by technicians (some increased time to clean the gastrointestinal tract) and pathologists (increased organ weights and vacuolation in the liver). Importantly, data from studies in non-fasted rats has not been questioned within regulatory submissions. Consequently, the results of the studies demonstrate that there is no evidence or scientific justification for fasting of animals on toxicology studies as standard, for any of the procedures for which this is commonly applied (before blood sampling or necropsy).

## 6. Discussion

Before any potential new medicine can be administered to humans, or new chemical marketed, their safety must be adequately assessed, which almost always includes some in vivo tests in rodent and/or non-rodent and/or relevant environmental species. The conduct of safety studies in animals is highly regulated within many regions to ensure optimization of animal welfare. Although the studies are largely standardized, this does not preclude the need to continuously reassess and challenge the design and implementation of such studies to ensure they are performed to the most up-to-date scientific knowledge and incorporate innovative technologies. This can refine animal use and improve scientific outcomes through improvements to technical procedures and/or adjustments to study designs.

Animals involved in metabolism and toxicology studies undergo many different procedures over the course of the study or their lifetime. Some of these procedures will be short in duration and of mild severity, such as dosing and blood sampling, whilst surgical interventions or multiple procedures to collect various study data may have potential for higher levels of suffering, pain or distress. Oftentimes other changes in environmental conditions (housing and access to food) are linked to these procedures, which can increase levels of stress that individual animals experience. This article highlights a number of refinements that can be adopted to reduce the time that an animal is separated from companions, with the recognition that social housing generally provides the best enrichment opportunity for the laboratory species used within toxicology packages. These can be simple changes via shorter blood sampling protocols if microsampling is adopted, or changes in technologies or equipment for collection of data from group housed animals [24,25,26,27]. Importantly, the data collected from group housed animals is the same or better quality and variability than historical data from animals housed individually. The topic of whether fasting is required before various procedures can also have implications linked to housing conditions; foraging for food is an activity which provides opportunities for rats, mice and NHPs to interact with cage/pen mates, occupies time, and provides structure within waking hours. Dogs and minipigs learn to anticipate the cues and timings for feeding and absence of food may be another source of stress. This can also lead to aggression; fasting has been shown to increase incidences of aggression in group housed mice [34] and an anecdotal increase in the incidence of fighting and bite wounds has been noted at some facilities when NHPs were fasted. Fasts of 16–18 h have been shown to decrease rat bodyweights by up to 7% compared to pre-fast bodyweights [34,35] and to affect other physiological and biochemical processes. Therefore, the need for fasting before clinical pathology bleeds should be reviewed for rats and for all species prior to other common toxicology procedures such as necropsies and oral dosing, ideally moving towards a harmonized approach across facilities for non-fasted animals as standard.

## 7. Concluding Remarks

The development of new technologies and adoption of best-practice approaches to laboratory animal housing and study procedures has generated a series of refinements that can be incorporated into animal studies throughout toxicology packages at all stages and for all species. Continued sharing of experience and data within the toxicology community promotes the advantages to animal welfare and study data that these refinements create, highlighting opportunities that can benefit greater numbers of animals and studies across the world in the future.

## Figures and Tables

**Table 1:** Serial microsampling of satellite animals vs composite microsampling of main test animals.

Animals/Group/Sex	Blood Sampling Timepoint						Total Number of Microsamples per Animal
	1	2	3	4	5	6	
Serial microsampling of satellite animals							
Animal 1	√	√	√	√	√	√	6
Animal 2	√	√	√	√	√	√	6
Animal 3	√	√	√	√	√	√	6
Composite microsampling of main test animals							
Animal 1	√			√			2
Animal 2	√			√			2
Animal 3	√				√		2
Animal 4		√			√		2
Animal 5		√			√		2
Animal 6		√				√	2
Animal 7			√			√	2
Animal 8			√			√	2
Animal 9			√				1
Animal 10				√			1

Serial blood samples obtained from groups of satellite animals designated for TK blood sample collection only. Each individual is microsampled at all time points, thereby providing exposure data on an individual animal basis. If larger samples (non-microsamples) were required, more animals would be needed. Composite blood samples obtained from main study animals designated for toxicity assessment (no requirement for satellite animals). Blood samples distributed across all animals within the group to provide overall exposure for the population. If larger samples (non-microsamples) were required, satellite group(s) using additional animals would be needed.

**Table 2:** Summary of hematology and clinical chemistry results in male Sprague Dawley rats.

Food Status	Statistic	Hematology Parameters			Clinical Chemistry Parameters				
		**Hb**	**RBC**	**Hct**	**Glucose**	**Trig**	**ALT**	**AP**	**Urea**
		**g/dL**	**×12^12^/L**	**L/L**	**mmol/L**	**mmol/L**	**iU/L**	**iU/L**	**mmol/L**
Fasted	*n* ^1^	17	17	17	20	19	19	18	20
	Adjusted mean	15.1	8.05	0.448	8.65	0.75	60	178	6.0
	Standard error	0.1	0.08	0.003	0.28	0.09	2	5	0.2
Non-fasted	*n* ^1^	17	17	17	20	20	20	20	20
	Adjusted mean	14.6	7.75	0.438	11.55	1.69	79	208	4.9
	Standard error	0.1	0.08	0.003	0.28	0.08	2	5	0.2

^1^*n* = 10 rats with two samples per rat. Sample numbers < 20 are due to clotted samples. Hb, hemoglobin; RBC, red blood cell count; Hct, hematocrit; Trig, triglycerides; ALT, Alanine Transaminase; AP, Alkaline Phosphatase. Although the standard panel of clinical pathology and hematology parameters were collected, for brevity only the parameters likely to be affected by fasting/associated reduction in water intake are presented, along with some additional liver and kidney markers.

**Table 3:** Summary of clinical pathology data from male Cynomolgus Macaques (aged under 60 months).

Site	Statistics	Glucose	Trig	ALT	AST	AP	Alb	TP
Food Status		mmol/L	mmol/L	U/L	U/L	U/L	g/L	g/L
(Animal Origin)								
UK Non-fasted (Mauritia or Asia)	*n*	257	257	257	257	257	257	257
	Mean	5.216	0.482	39.4	37.9	596.9	44.22	74.41
	SD	1.238	0.217	12.2	9.3	175.3	3.47	4.11
	C of V	0.23	0.45	0.31	0.25	0.29	0.08	0.06
North America Fasted (Asia)	*n*	447	447	447	447	447	447	447
	Mean	3.70	0.574	51.1	51.9	560.8	44.2	73.4
	SD	0.91	0.195	18.8	25.4	167.8	3.00	4.7
	C of V	0.25	0.34	0.37	0.49	0.30	0.07	0.06

SD, standard deviation; C of V, coefficient of variation; Trig, trigycerides; ALT, Alanine Transaminase; AP, Alkaline Phosphatase; Alb, Albumin; TP, total protein. Although the standard panel of clinical pathology and hematology parameters were collected at each site, only the parameters where the same analysis method were used are presented. Samples were taken from control animals pre-trial and during the toxicology study in which it was used in 2019 or 2020; *n* represents the number of individual NHPs (mean values are shown for each animal).

**Figure A1:**
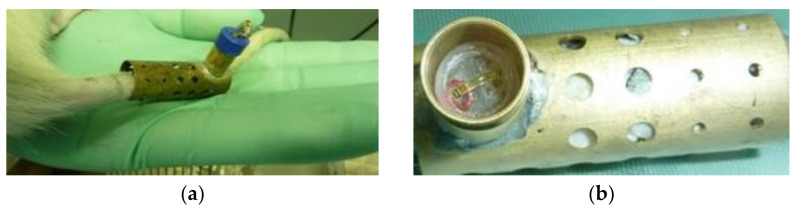
Photos of the pin-port tail cuff in-situ: (**a**) during study data collection periods; (**b**) close-up view of the U-shaped loop during recirculation phases (following surgery or between data collection sessions).

## Data Availability

Not applicable.

## Appendix A

Surgical details and technical information regarding the BDC rat model (to accompany Section 3 of the main text):

Rats undergoing surgery are anesthetized with isoflurane in oxygen and given carprofen for pain relief along with the antibiotic cefuroxime. The bile duct is exposed through a mid-line incision and a cannula inserted proximally in the direction of the liver and secured with ligatures. The distal end of the cannula is exteriorized via the tail. A second cannula is inserted into the duodenum. The two catheters exteriorized from the tail are attached to a port protected by a modified tail cuff. A U-shaped loop is placed in the socket so that bile from the bile ducts flows into the duodenum (Figure A1b).

At the start of the experimental phase of the study, the rat is placed within a metabolism cage and the U-shaped loop is removed from the tail cuff adaptor. External cannulae for collection of bile and reinfusion of bile salts are fed through a protective coiled stainless-steel tube connected to a swivel device and the extensions fitted to the port vacated by the loop (Figure A1a). The apparatus is designed so that the cannulae for collection and reinfusion cannot be switched in error. The tube and swivel are designed to allow freedom of movement of the animal whilst maintaining the patency of the cannula. Artificial bile salts are infused continuously through the duodenal cannula during the sample collection period.

At the completion of each experimental phase, animals are removed from the metabolism cage and external cannulae and swivels removed. A naïve U-shaped loop can be placed into the adaptor and the animals returned to group housing.

Animals were subjected to the following sampling collection and respite schedule during the validation study (Table A1):

**Table A1:** Study design for the pin-port BDC validation study.

Day	Activity	Housing
0	Surgery	-
1–4	Recovery from surgery	Grouped ^1^ in home cage
4–8	Oral dose and sample collection period 1	Single housed in metabolism cage
8–11	Respite between recording sessions	Grouped in home cage
11–14	Intravenous dose and sample collection period 2	Single housed in metabolism cage
14–19	Respite between recording sessions	Grouped in home cage
19–20	Sample collection period 3	Single housed in metabolism cage
20–22	Respite between recording sessions	Grouped in home cage
22–23	Sample collection period 4	Single housed in metabolism cage
23–25	Respite between recording sessions	Grouped in home cage
25–26	Sample collection period 5	Single housed in metabolism cage

^1^*n* = 3.
